# A novel Lab-on-Fiber Radiation Dosimeter for Ultra-high Dose Monitoring

**DOI:** 10.1038/s41598-018-35581-3

**Published:** 2018-12-14

**Authors:** Giuseppe Quero, Patrizio Vaiano, Francesco Fienga, Martino Giaquinto, Valentina Di Meo, Georgi Gorine, Pierluigi Casolaro, Luigi Campajola, Giovanni Breglio, Alessio Crescitelli, Emanuela Esposito, Armando Ricciardi, Antonello Cutolo, Federico Ravotti, Salvatore Buontempo, Marco Consales, Andrea Cusano

**Affiliations:** 1 Optoelectronics Group - Department of Engineering, University of Sannio, I-82100 Benevento, Italy; 2 Istituto Nazionale di Fisica Nucleare (INFN) - Sezione di Napoli, I-80126 Napoli, Italy; 3 Istituto per la Microelettronica e Microsistemi, Consiglio Nazionale delle Ricerche, I-80131 Napoli, Italy; 4 European Organization for Nuclear Research (CERN), 1211 Genève, Switzerland; 5 Ecole Polytechnique Federale de Lausanne (EPFL), Lausanne, Vaud Switzerland; 6 University of Napoli Federico II, Department of Physics, I-80126 Napoli, Italy; 7 University of Napoli Federico II, Department of Electronical Engineering, I-80125 Napoli, Italy

## Abstract

In this work, we report on the first demonstration of Lab on Fiber (LOF) dosimeter for ionizing radiation monitoring at ultra-high doses. The new dosimeter consists in a metallo-dielectric resonator at sub-wavelength scale supporting localized surface plasmon resonances realized on the optical fiber (OF) tip. The resonating structure involves two gold gratings separated by a templated dielectric layer of poly(methyl methacrylate) (PMMA). Two LOF prototypes have been manufactured and exposed at the IRRAD Proton Facility at CERN in Geneva to 23 GeV protons for a total fluence of 0.67 × 10^16^ protons/cm^2^, corresponding to an absorbed dose of 1.8 MGy. Experimental data demonstrated the “radiation resistance” feature of the LOF devices and a clear dependence of the reflected spectrum versus the total dose, expressed by a cumulative blue-shift of ~1.4 nm of the resonance combined with a slight increase of 0.16 dBm in the reflected spectrum. The numerical analysis carried out to correlate the experimental results with the dimensional and physical properties of the resonator, expected to be tightly connected to the absorbed dose, suggests that the main phenomenon induced by exposure to proton beam and able to explain the measured spectral behavior is the reduction of the PMMA thickness, which is also consistent with past literature in the field. Preliminary results demonstrated the potentiality of the proposed platform as dosimeter at MGy dose levels for high energy physics experiments.

## Introduction

The High Energy Physics (HEP) community is currently discussing the possible upgrades of today’s Large Hadron Collider (LHC), in order to schedule a roadmap for the construction of the next generation HEP experiment. Along with the Compact Linear Collider (CLIC^1^) and the High Energy Large Hadron Collider (HE-LHC^2^), the Future Circular Collider (FCC) is the most ambitious of the proposed machines, with its 100 km long tunnel, being designed to reach hadron-hadron collisions at 100 TeV, about 8 times higher than in today’s LHC^3^. Such new synchrotron ring will allow to expand the current energy and luminosity frontiers, pushing towards novel discoveries in the particle physics field. Along with the civil engineering challenges of building a 100 km long tunnel, several other technological challenges are being considered in order to be able to build and operate such a poweFul machine. Among these challenges, the very harsh radiation environment, evaluated to be several orders of magnitude higher than in the current LHC, will require new dedicated technologies for the detectors and electronics.

The impact of cumulative radiation damage to materials (such as semiconductors) is commonly measured in term of Total Ionizing Dose (TID) and Displacement Damage (DD). The TID is proportional to the ionizing energy released in the material and is expressed in J/Kg or Gray [Gy]. The DD is instead proportional to the number of particles crossing the material and is expressed in particles/cm^2^ [Ф]. For semiconductor material, the normalized 1 MeV neutron equivalent fluence [n_1MeV_/cm^2^] is instead commonly used.

In the LHC, the total Dose and Fluence, cumulated over 10 years of operation, are expected to be about 100 kGy and >10^15^ particles/cm^2^ respectively (in the LHC experiments). In order to keep these radiation levels under constant monitoring, the Radiation Monitoring (RADMON) dosimeters are employed in different sections of the tunnel and its experiments (PH-RADMON, targeting the needs of LHC experiments^4^ and LHC-RADMON for the tunnel area^5^).

In the case of the FCC, due to an increased collision energy (from 14 TeV to 100 TeV) and rate (from 0.85 GHz to 32.4 GHz)^3^, radiation levels are expected to be several orders of magnitude higher than today’s LHC, exceeding several tens of MGy (with >10^17^ particles/cm^2^) inside the FCC experiments^6^, and tens of kGy (with >10^15^ particles/cm^2^) in certain sections of the FCC tunnel itself^7^.

**Figure 1:**
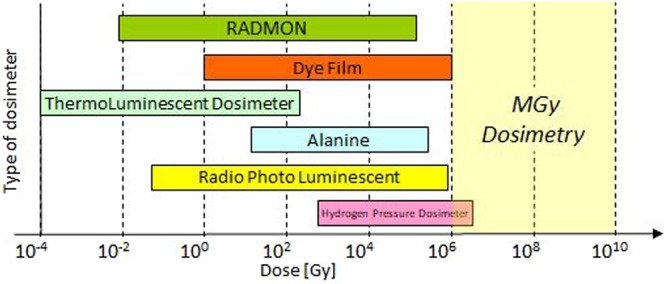
Different active and passive technologies for TID dosimetry available on the market are shown with respect to their monitoring range^60^. The highlighted and empty area in the Ultra High Dose range indicates the target area of the technology presented in this paper.

Figure 1 shows the integration ranges of the RADMON as well as other types of dosimeters that are today commonly used. Special focus is made on the “MGy range” expected for the FCC experiments, where none of these technologies are capable of properly function, because of saturation problems and/or extensive and irreversible radiation damage.

These noticeable limitations have triggered an extensive research for new technologies^8^, and while for the ultra-high fluence monitoring devices based on metallic thin-films are currently under research at CERN and are showing promising results^9^, no solutions have yet been proposed for an ultra-high dose monitoring technology that could be coupled to the PH-RADMON extending its range of detection across and beyond the MGy range.

In the present work, we report the first proof of principle of an alternative technology for the ultra-high dose monitoring based on the LOF technology^10–12^. The main characteristics, working principle, and target operational range of the work here presented are summarized in Table 1 and compared with the dosimeters found in the PH-RADMON.

**Table 1:** Operational range of current PH-RADMON devices for the LHC experiment^4^ as compared with the performance required (after 10 years of operation) from the new Ultra High Fluence and Dose dosimetry for the future HL-LHC upgrade and FCC^4,7,60^.

	LHC		FCC	
	Diodes LBSD/BPW^4^	RadFETs LAAS/REM^4^	Ultra High Fluence Monitor^8^	Ultra High Dose Monitor^[this work]^
Type of Device	p-i-n photodiode	Thick oxide MOSFET	Thin metal film Resistors	Metallo/dielectric resonator on Fiber Optic
Working Principle	Increase of diode’s forward current	Shift of transistor threshold voltage	Increase of film resistivity	Shift of resonance frequency
Radiation Interaction	Displacement Damage in Silicon	Charge trapping in gate oxide	Displacement Damage in Metal*	Compression of PMMA layer due to absorbed dose*
Target Max Radiation (over 10 years)	Tunnel: 1.0 × 10^11^ n_1MeV_/cm^2^ Experiments: 1.0 × 10^15^ n_1MeV_/cm^2^	Tunnel: 1 kGy Experiments: 100 kGy	Tunnel: 1.0 × 10^13^ n_1MeV_/cm^2^ Experiments: 2.8 × 10^16^ n_1MeV_/cm^2^	Tunnel: 100 kGy Experiments: 90 MGy
Device Operating Range	LBSD: 1.0 × 10^8^÷2.0 × 10^12^ BPW: 2.0 × 10^12^÷2.0 × 10^15^	LAAS: 0.01 Gy÷10 Gy REM: 10 Gy÷100 kGy	From 10^15^ n_1MeV_/cm^2^	From 100 kGy

(*effects being investigated in ongoing activities).

This paper is structured as follows: Section “Optical fiber dosimeters” gives an overview of the existing OF dosimeters; Section “Lab on Fiber technology: a promising approach for MGy dosimetry” provides a specific description of the proposed innovative dosimetry technology including the LOF probe design, fabrication and characterization, as well as numerical simulations, based on finite element analysis, predicting the effects of perturbations of different parameters on the LOF spectral response; Section “Irradiation Tests at CERN” deals with the irradiation tests, providing details about the CERN IRRAD facility and the experimental results recorded with LOF probes; Section “Data analysis and discussion” focuses on the processing of the experimental data and comparison of LOF performance versus the numerical simulation and literature; Section “Conclusions and future works” gives our conclusions concerning the first application of LOF technology for dosimetry at ultra-high dose levels and a view on the planned future works to further investigate these innovative dosimetry technique.

### Optical fiber dosimeters

In order to overcome the impossibility to monitor ultra-high dose levels with the current technologies, in the last years a great effort has been made to demonstrate the applicability of OFs in this field^13,14^. The inherent properties of OFs, including the small size, low mass, immunity to electromagnetic interference, possibility of multiplexing and real-time, continuous and remote measurement^15^, represent unrivaled advantages for OF-based dosimeters^16^. According to the role played by the fiber within the measurement system, OF dosimeters can be classified, into intrinsic and extrinsic architectures. In intrinsic configurations, where the OF acts both as the sensing and the light-guiding components^16^, the main effects exploited are the radiation-induced attenuation (RIA)^17–19^ and the radiation-induced changes in the refractive index (RI) of the fiber^20^, which also led to the application of Fiber Bragg Gratings (FBGs) and Long Period Gratings (LPGs), notoriously sensitive to changes in the fiber RI, as dosimeters^21–26^. Differently, in extrinsic OF dosimeters, the OF serves only as a guide of the light travelling to and from a material sensitive to the radiation. These are the majority and, among their basic exploited principles, they can be recalled the scintillating materials, thermo-luminescence and optically stimulated luminescence^27–35^. It is worth noting that an important contribution to the development of OF dosimeters has been provided by the use of poly(methyl methacrylate) (PMMA) as sensing element^13,16^.

The performance of OF dosimeters reported so far, including dose sensitivity and operation ranges, is strongly influenced by the fiber chemical composition, radiation environment and manufacturing process^36^. Indeed, in the case of FBGs written in pure-silica fibers, which exhibit no change of the Bragg wavelength, as well as of the amplitude and the shape of the spectra, at a dose of 100 kGy^25^, it has been demonstrated that the dose sensitivity and operation range can be significantly modified by doping the fibers, achieving limits of saturation even higher than 200 kGy^22,37^. Resonance wavelength shifts definitely larger than those measured in FBGs have been observed in turn around points (TAP) LPGs (up to 65 kGy^24^) and chiral LPGs (up to 100 kGy without complete saturation^23^). In this context, a clear definition of the limits of operation is not yet possible, as the experiments reported so far are few, as well as the exploited dose rates scenario, which can have a major influence on the response of the sensor and its saturation point. A notable drawback for these dosimeters is the remarkable temperature sensitivity, especially in TAP-LPGs. Moreover, a large number of these approaches, especially those based on the measurement of the optical signal intensity, have the drawback of requiring a highly stable set-up, which is not always available in a hostile radiation environment^13,16^.

In order to meet the abovementioned needs and to overcome the limitations suffered by the majority of OF-based approaches, especially in terms of operational range and saturation levels, as well as influence of temperature and issues related to intensity-based measurements, in this work, we propose a new extrinsic OF dosimeter for ultra-high dose radiation, based on the so-called LOF technology^10–12^.

The key idea at the basis of LOF technology is to transform a ‘simple’ OF into a miniaturized and multifunctional platform for specific applications through the integration of functionalized materials and components defined at micro or nanoscale^10,12,38,39^. After devoting a great effort to solve manufacturing issues, our attention has been focused on the development of LOF prototypes for specific applications, mainly (but not limited to) chemical and biological sensing^12,39,40^.

Here, the proposed LOF platform consists in a metallo-dielectric resonator realized on the OF tip (OFT)^38,39^ including a patterned PMMA layer covered by a thin gold overlay. Our expectation is to reduce saturation effects taking advantage from the large set of radiation sensitive materials involved in, while wavelength encoding detection allows robust measurements against intensity fluctuations along the whole optical chain.

Preliminary experimental results reported here demonstrated indeed the effectiveness of the proposed OF device as potential dosimeter at MGy dose levels, paving the way for its future application in HEP experiments.

### Lab on Fiber technology: a promising approach for MGy dosimetry

Among the different configurations embraced by the LOF concept, the OFT represents an attractive platform for remote sensing applications, thanks to its inherently light coupled micro-sized active area, joined with the faculty of integrating complex micro- and nano-metric structures made of dielectric and metallic materials supporting resonant light trapping effects. The principle of operation of this kind of devices is based on the sensitivity of such resonant phenomena to the optical and geometrical variations induced from the surrounding.

**Figure 2:**
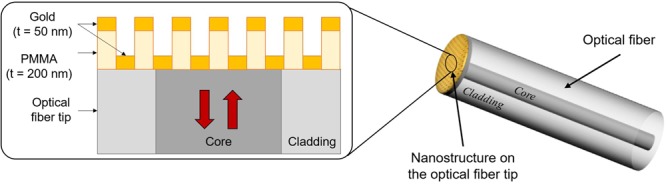
Cross section view of the LOF resonator realized on the OFT.

Based on a previous demonstration of LOF device carried out by the Unisannio group^38^, the present work deals with the realization of a metallo-dielectric resonator on the OFT sensitive to radiation. In particular, the LOF structure can be regarded as the superimposition of two metallic gratings separated by a dielectric layer (Fig. 2).

By illuminating the metal-dielectric structure in ‘out-of-plane’ configuration, localized surface plasmon resonances may be excited by the phase-matching conditions between the scattered components and the modes supported by the hybrid structure^38^. The spectral response arising from these resonant phenomena is sensitive to variations of both optical properties and size of the platform components. These include real and imaginary parts of the RI of adopted materials, as well as lattice period, holes radius, metal and dielectric thickness. The dielectric layer, in particular, is one of the constituent elements able to play a fundamental role in the design and tailoring of the spectral features of a LOF device for dosimetry application. Among dielectrics, polymers are recognized as highly sensitive to radiations^41^. Within this class of materials, the attention has been focused here on the PMMA because of the well-known effects induced by proton beams on its optical and geometrical properties^42–45^ and since it exhibits a RI close to 1.5, i.e. higher than that of the OF, creating a condition that promotes the excitation of spectral resonances^38^.

### LOF probe design

**Figure 3:**
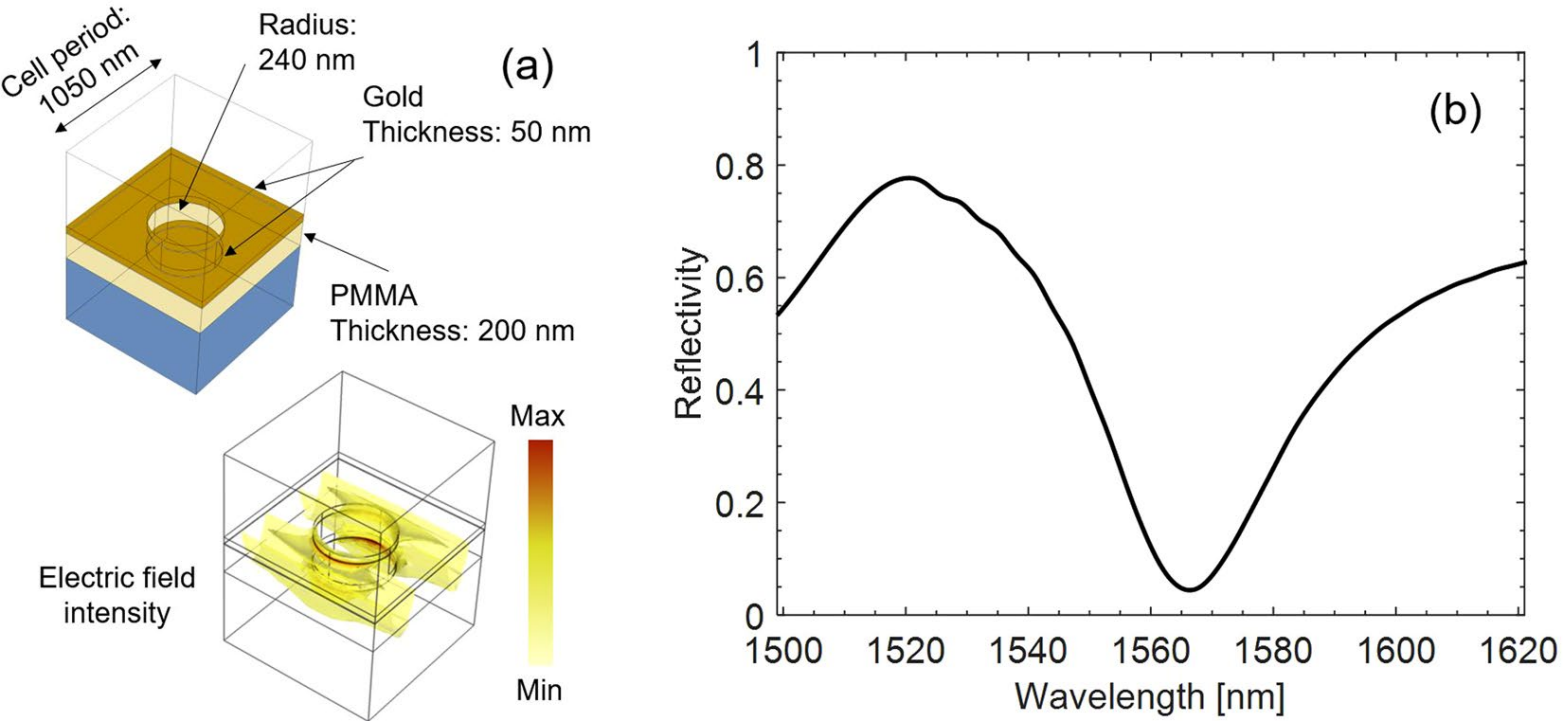
(**a**) Schematic of the LOF nanostructure (unit cell) and electric field distribution in correspondence of the resonance wavelength (λ_res_∼1566 nm); **(b)** numerical reflected spectrum.

The LOF probe design is based on the use of the finite element method provided by the Comsol Multiphysics – RF module^46^. The design parameters, including a holes radius of 240 nm, grating period of 1050 nm, PMMA and gold thickness of 200 nm and 50 nm, respectively (Fig. 3a), have been chosen in order to set the resonance wavelength in the operating range of standard single mode fibers, enabling the use of robust and reliable commercially available optoelectronic interrogation units. As shown elsewhere^47,48^, the exploitation of the crystal translational symmetries can reduce the computational domain to the unit cell (given by one period of the structure), terminated with Floquet boundary conditions, placed two-by-two in the opposite walls. The resulting structure supports a transverse-electromagnetic wave emulating the normally incident plane-wave. This kind of excitation was preferred to a proper fiber mode with Gaussian profile to simplify the numerical simulations^38^. The RI data used for modeling gold in the IR region were taken from ref.^49^, for silica from ref.^50^, while for PMMA from ref.^51^. The numerical reflected spectrum of the LOF structure in a normalized scale exhibits a high reflectance value (higher than 0.6) interrupted by a resonance dip centered at λ_res_∼1566 nm with a Q-factor of ∼47 (Fig. 3b).

The exposure to proton beam could involve the modification of one or more parameters of the LOF structure, affecting both its optical properties (variations of the real or imaginary part of the materials) and geometry (such as the thickness of the layers or the dimensions of the holes and their period).

Depending on the class of materials used to build each single component of the structure, the radiation effects on the LOF device can be essentially divided into three main groups: effects occurring on the polymer, on the metal and on the fiber itself (see Table 2).

**Table 2:** Resume of the main radiation effects reported in literature occurring in the classes of materials used to build the LOF device.

Material	Phenomenon reported	References
PMMA	Increase of the real part of the RI as the result of compaction of the irradiated PMMA layer (densification). Increase of the propagation losses.	^42– 45^
Metal	Increase of metal resistivity with increasing particle fluence.	^64– 66^
Fiber glass	Increase of the imaginary part of the RI (radiation-induced attenuation). Increase of the real part of the RI (radiation-induced RI variation).	^20, 25^

With regards to the polymeric layer, it is well known that proton radiation causes several changes in the PMMA, including an increase of the propagation losses^42,44^ and phenomena of compaction and densification^42–45^. The consequences on the PMMA of these phenomena in terms of optical properties are an increase of the imaginary part of the RI (k_PMMA_), due to the enhanced losses, and an increase of the real part (n_PMMA_) due to the densification. Moreover, compaction may modify the geometry of the PMMA layer along both the vertical direction, implying a reduction in thickness (t_PMMA_), and the horizontal one, causing an enlargement of the holes radius (r_holes_).

Concerning the metallic layer, experimental results recently reported elsewhere^8^ showed that thin layers of certain metals, specifically copper, with 500 and 1000 nm thickness, when exposed to proton beam exhibit a positive variation of resistivity with increasing particle fluence due to the accumulated displacement damage.

A variation in resistivity, as it implies a reduction in the concentration of free charge carriers, causes also a change in the real (n_gold_) and imaginary part (k_gold_) of the RI of the material^52^. In particular, an increase of n_gold_ and a decrease of k_gold_ are expected^53^. Irradiations of 20 µm layer of gold with fast neutron up to 2.2 × 10^19^ n/cm^2^ showed an increase of the gold resistance with the irradiation dose, at fixed temperature^54^. Nevertheless, a deeper understanding of the effects of very high particle fluence, in the range of interest of future HEP experiments, on nanometric metal films including the physical mechanisms leading to an increase of the resistivity is necessary.

Actually, it must be said that the interaction with radiations leads to a number of chemical and/or physical changes also in the fiber glass, including the creation of preferential absorption in a broad spectral range, well-known as RIA^36^. Indeed, the enhancement of the imaginary part of the silica RI (k_fiber_) responsible of the absorption is also associated to an increase of the real part of the RI (known as radiation-induced RI variation), according to the Kramers-Kronig relations^25^. Such phenomena, as stated before, are the basis of intrinsic OF dosimeters.

Once identified the effects that ionizing radiations may induce on the optical and geometric parameters of the LOF device, numerical simulations have been carried out using the aforementioned RF module to evaluate the consequences produced by the variation of each single parameter of the structure on the reflected spectrum, keeping the others fixed.

**Figure 4:**
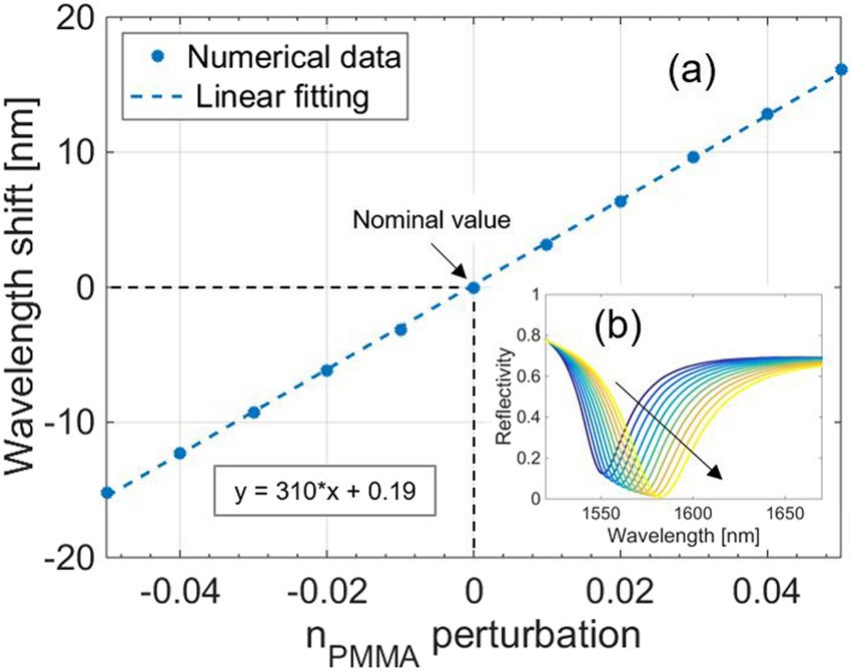
(**a**) Numerical simulations predicting the effects of a perturbation of n_PMMA_ on the LOF resonance wavelength. Black dashed lines refer to nominal value of n_PMMA_. The inset box indicates the equation of the linear fitting. **(b)** Whole spectrum evolution determined by the variation of n_PMMA_. The black arrow indicates the variation direction of the parameter.

In particular, the results of the simulations of a perturbation of n_PMMA_, performed with steps of 10^−2^, showed a linear dependence of the resonance wavelength from the changes of n_PMMA_ with a positive slope of 310 nm/refractive index unit (RIU) (Fig. 4a). Furthermore, the evolution of the reflected spectrum occurring when n_PMMA_ increases (Fig. 4b) shows a slight decrease of the reflectivity outside the resonant dip (hereafter called baseline).

**Figure 5:**
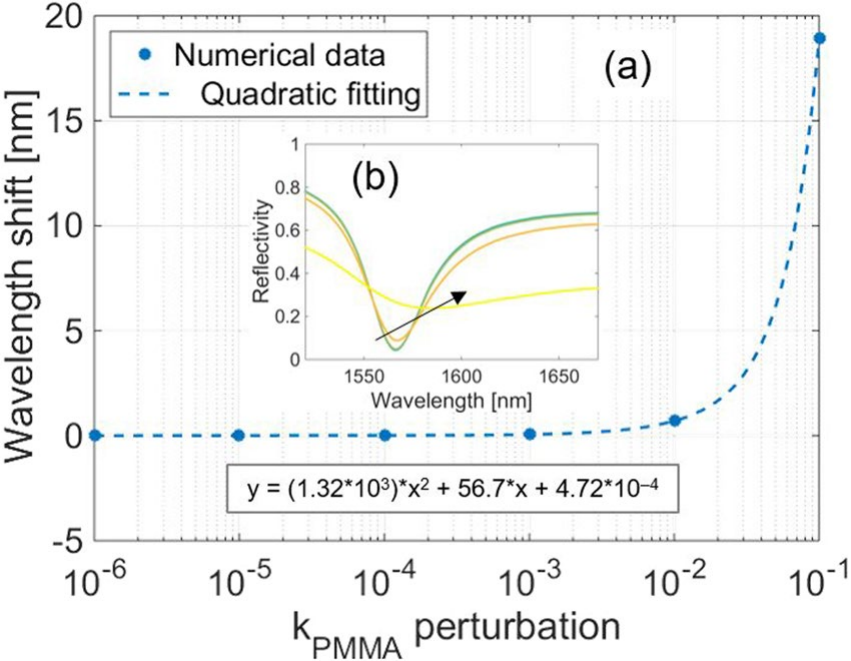
(**a**) Numerical simulations predicting the effects of a perturbation of k_PMMA_ on the resonance wavelength. Logarithmic scale is used for the x-axis. The inset box indicates the equation of the quadratic fitting. **(b)** Whole spectrum evolution determined by the variation of k_PMMA_. The black arrow indicates the variation direction of the parameter.

Differently, a perturbation of k_PMMA_ has no consequences on the resonance wavelength until a value of 10^–2^, beyond which a fast red shift following a quadratic behavior is expected (Fig. 5a). Such large perturbations of k_PMMA_ also cause a strong deformation of the resonant dip, clearly visible in Fig. 5b.

**Figure 6:**
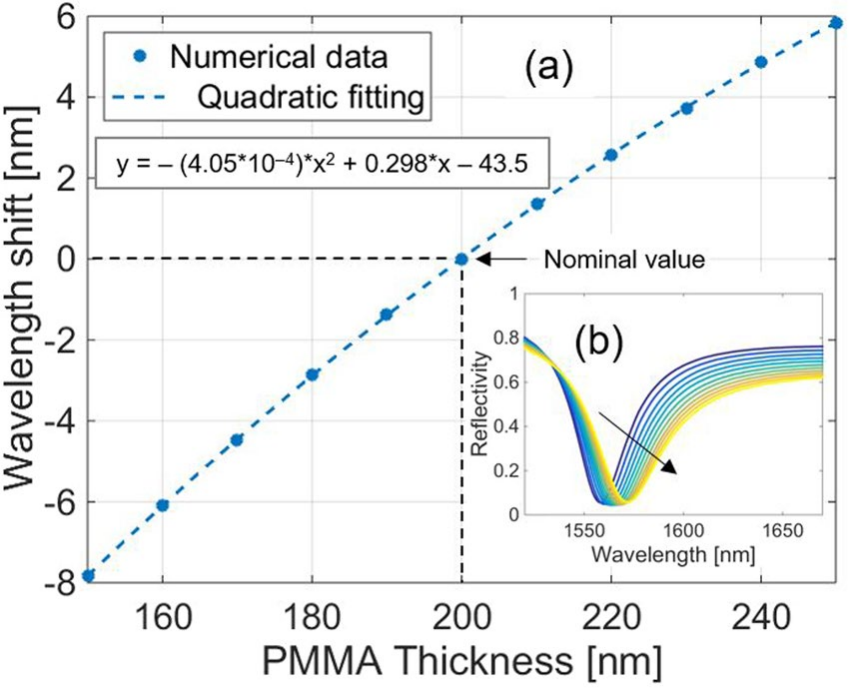
(**a**) Numerical simulations predicting the effects of a variation of t_PMMA_(with steps of 10 nm) on the resonance wavelength. Black dashed lines refer to nominal value of t_PMMA_. The inset box indicates the equation of the quadratic fitting. **(b)** Whole spectrum evolution determined by the variation of t_PMMA_. The black arrow indicates the variation direction of the parameter.

As regards the thickness of the PMMA layer t_PMMA_, its change leads to an increasing quadratic variation of the resonance wavelength (Fig. 6a), while the baseline is a decreasing t_PMMA_ function, as shown in Fig. 6b.

The effect of the other parameters of the structure influenced by radiation on the LOF platform spectrum predicted by numerical simulations are reported in detail in the Supplementary Information (Section S1).

Table 3 summarizes the radiation induced changes in the properties of the different materials constituting the LOF platform according to previous literature and includes the sensitivity analysis to these properties expressed in terms of resonant wavelength and reflectance baseline (far from the resonance) provided by our numerical investigation. Both the sign and the range of variation are shown, where present. When the dependence of the spectral observable from a given parameter is not linear, the sensitivity has been calculated by linearizing the characteristic curve around the nominal value of the parameter itself. Since the values reported in the literature refer to different types of radiation, energy levels, doses, fluences, operational environments spectral ranges, it is clear that the reported table is purely indicative.

**Table 3:** Sensitivity analysis. The signs reported in the second column follow the legend: “ + “ for a positive change, “−“ for a negative change. The reflectance baseline sensitivity is evaluated on the normalized reflectance in the range 0–1.

Material Property	Sign	Variation reported in literature	LOF structure	
		Range	λ_RES_ Sensitivity	Baseline Sensitivity
Δn_PMMA_	+	n_PMMA,nom_ = 1.490 @ 633 nm [refs^42,44^] Δn_PMMA_ = 3.1 ÷ 4.8·10^–3^ with 1 ÷ 2 MeV protons (fluence of 2 ÷ 5·10^13^/cm^2^) [refs^42,44^]	+ 310 nm/RIU	–0.375 RIU^-1^
Δk_PMMA_	+	k_PMMA,nom_ = 5.1·10^−6^ [ref.^42^] Δk_PMMA_ = 1.6 ÷ 3.5·10^−6^ with 350 keV protons (fluence of 2 ÷ 8·10^14^/cm^2^) @ 635 nm [ref.^42^]	+ 70 nm/a.u.	–5.21 a.u.^-1^
Δt_PMMA_	−	t_PMMA,nom_ = 1 mm ÷ 3 mm [refs^43–45^] Δt_PMMA_ = 160 nm ÷ 2.4 µm with 350 keV ÷ 2 MeV protons (fluence of 2 ÷ 5·10^13^/cm^2^ 1·10^15^/cm^2^) [refs^43–45^]	+ 0.136 nm/nm	−0.001 nm^−^^1^
Δr_holes_	+	Data Not Available	+ 0.183 nm/nm	+0.002 nm^−^^1^
Δn_gold_	+	Data Not Available	−1.9 nm/RIU	−0.18 RIU^−^^1^
k_gold_	−	k_gold,nom_ (λ = 1566 nm) = 9.91 [ref.^49^] Δk_gold_ = 3.43 with neutrons (fluence of 2.2·10^19^/cm^2^) [ref.^53^]	−7.58 nm/a.u.	−0.03 a.u.^−^^1^
Δn_fiber_	+	n_fiber,nom_ = 1.453 ÷ 1.455 @ 1566 nm [ref.^20^] Δn_fiber_ = 2 ÷ 4·10^−^^4^ @ 1566 nm with γ irradiation [ref.^20^]	+ 721 nm/RIU	−0.006 RIU^−^^1^
Δk_fiber_	+	k_fiber,nom_ = 1.29·10^−^^10^ @ 1550 nm [ref.^67^] Δk_fiber_ = 3.9·10^−^^2^ with γ irradiation, 100 kGy (1 Gy/s) [ref.^25^]	−39.1 nm/a.u.	−7.4 a.u.^−^^1^

### Probe Fabrication

**Figure 7:**
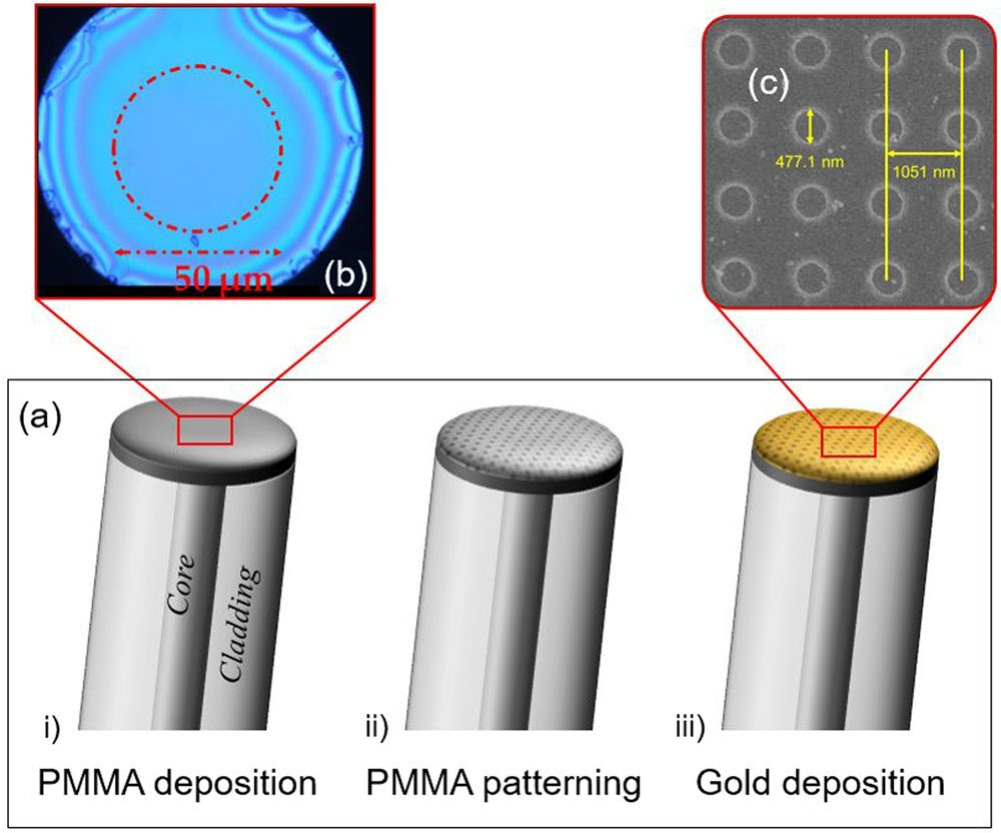
(**a**) Schematic representation of the technological steps used in the probe realization process: i) polymeric overlay deposition, ii) OFT exposure to electron beam and polymer development, iii) gold superstrate deposition. **(b)** Optical microscope image of an OFT after the deposition of the PMMA layer, showing a uniform region with a diameter of about 50 μm around the fiber core. **(c)** SEM image showing the top view of the metallo-dielectric nanostructure realized on the OFT.

The realization of the 2D hybrid metallo-dielectric nanostructure consists of three main steps^38^, as schematically explained in Fig. 7a: i) polymeric overlay deposition onto the OFT, ii) OFT exposure to electron beam and polymer development, iii) gold superstrate deposition.

The uniform PMMA deposition on the OFT has been achieved by using a customized spin coater involving a rotating plate modified in order to host the OF. For the purpose, a standard single mode OF (Corning SMF-28) was adopted. After the OF housing in the drilled plate, the PMMA solution was spun upon it. As shown in Fig. 7b, the uniformity of the PMMA layer has been guaranteed in the central part of the OFT for a region of about 50 μm, definitely wider than the fiber core diameter. Furthermore, a careful combination between the spin-coater plate velocity (2000 rpm) and the rotation time (1 minute) allowed obtaining a PMMA thickness of about 200 nm, as dictated by the design.

After the PMMA overlay deposition onto the OFT, a periodic pattern has been obtained through Electron Beam Lithography (EBL). Following the design, a periodic structure consisting of a holes array with a period of ~1050 nm and a radius of ~240 nm has been realized in the PMMA layer using the RAITH 150 EBL system. The EBL process involved an electron acceleration voltage of 20 kV, a dose of 120 μC/cm^2^ and an electron beam aperture of 7.5 μm. After the PMMA electronic impression, the desired 2-dimensional (2D) array has been obtained by developing the polymeric layer.

The last realization step relies on the deposition of a 50 nm-thick gold superstrate onto the polymeric pattern realized by means of a conventional DC magnetron sputtering. To ensure the good uniformity of deposition, a dedicated holder has been designed and realized to keep the OFT perpendicular to the gold target.

### Morphological and optical characterization

After manufacturing, two prototypes of the proposed LOF platform (henceforth referred to as prototypes A and B) have been fabricated on two different fibers. A morphological characterization of the devices has been carried out by acquiring several scanning electron microscope (SEM) images of the structures (Fig. 7c).

**Figure 8:**
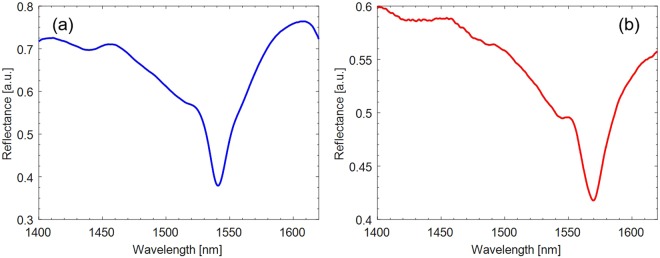
Experimental normalized reflectance spectrum of the 2D hybrid metallo-dielectric structure: **(a)** prototype A; **(b)** prototype B.

With the aim of evaluating the spectrum reflected by the LOF probes, an optoelectronic set-up comprising a broadband light source (with bandwidth 1200–1700 nm), a directional coupler and an optical spectrum analyzer (OSA), ANDO AQ6317C, with best wavelength resolution of 10 pm and dynamic range 60 dB, was adopted. The OSA was connected to a computer and controlled by a LabVIEW^TM^ plug-in. In order to compensate for the intensity fluctuations which may occur during the characterization, the LOF reflectance spectrum was calculated by normalizing the reflected spectrum with that reflected by a fiber-optic reference mirror, fabricated by depositing a 160 nm-thick gold film on the tip of a standard single-mode fiber. The experimental reflectance spectra of the LOF prototypes are shown in Fig. 8. Comparing the experimental and simulated spectra (Fig. 3b), slight differences in the exact location of the resonance wavelength and visibility can be noted. These discrepancies can be attributed to the fabrication tolerances influencing both the holes radius and period. Nonetheless, both LOF devices exhibited a resonance dip centered around 1550 nm, enabling the use of a compact optoelectronic interrogation unit to monitor the spectrum during the proton irradiation.

### Irradiation Tests at CERN

To test the response of the sensor to ultra-high levels of radiation, as the ones encountered in the future generation of particle accelerators, the first irradiation experiments have been performed at the high-energy proton irradiation facility (IRRAD), located at the East experimental area of the Proton Synchrotron (PS) accelerator at CERN^55^.

**Figure 9:**
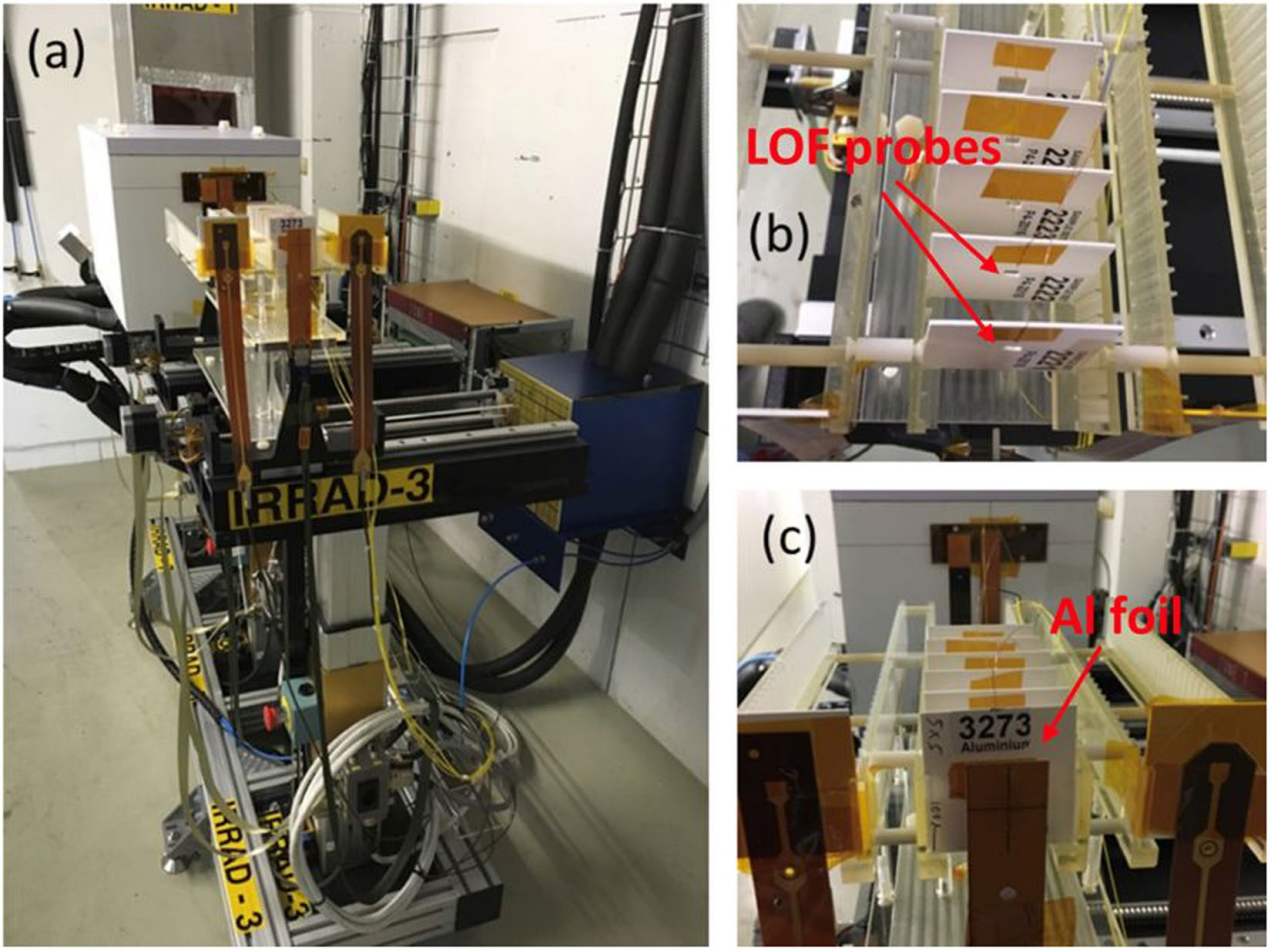
(**a**) OF samples placed on the irradiation table IRRAD3; (**b**,**c)** detail of the OF sensor mounted on the sample holders.

The PS delivers to IRRAD a Gaussian proton beam of 23 GeV with a typical size of 12 × 12 mm^2^, in spills of ~5 × 10^11^ p/spill, every 10 seconds on average. With such configuration, samples typically of 10 × 10 mm^2^ in size can be exposed to a fluence of 10^16^ p/cm^2^ in about 2 weeks, while smaller objects as the OF sensors described in this work, over the same period of time, can be irradiated up to higher particle fluence approaching the 10^17^ p/cm^2^. Concerning the samples, these are placed on remotely controlled tables that can be moved along the X-, Y-axis in order to precisely align them with the beam as shown in Fig. 9a. The tables are located in three different irradiation zones according to the nature of the samples to be irradiated. Due to the intrinsic “low-mass” of the OF samples, two prototypes of the proposed LOF dosimeter have been installed upstream the IRRAD facility on the table IRRAD3 in Zone 1^56^.

The intensity of the extracted proton spills is monitored using a Secondary Emission Chamber (SEC) device provided by PS beam instrumentation, while the profile of the proton beam, with a spill-by-spill resolution, is obtained by a custom-made Beam Profile Monitor (BPM)^57^. The calibration of this beam instrumentation, as well as the final total number of protons that crossed the sample (normalized per cm^2^) during the irradiation test, is obtained by means of activation measurements of thin Aluminium (Al) foils accurately inserted in front of the irradiated sample^58^.

The basic measurement performed on the 23 GeV proton beam is the determination of the proton fluence by evaluating the ^24^Na and ^22^Na activity in thin Al foils produced via dedicated nuclear reactions^59^. With these activation techniques it is possible to obtain fluence measurements with an accuracy of ±7%.

Taking into account that ionization is the main contribution to the energy loss of a charged particle and that its mean value, the stopping power (*dE/dx*), is given by the Bethe–Bloch law, it is possible to simply convert the fluence into the dose (Gy) deposited in thin samples, using the following formula:

$$D=K\cdot {(dE/dx)}_{m}\cdot \phi $$

where *φ* is the proton fluence expressed in particles/cm^2^, K = 1.602*·*10^−10^ is the scale factor and *(dE/dx)*_*m*_ expressed in MeV·cm^2^/g is the minimum ionizing energy loss rate. For 23 GeV protons it has value 1.664 MeV·cm^2^/g for Silicon (Si). Si was chosen as reference material for the absorbed dose conversion from measured particle fluence, since standard dosimetry for electronics is commonly calculated in Si. This choice is also justified by the fact that the energy loss of particles at energies of 23 GeV is about the same for any material^60^ (e.g.^61^ Al 1.615 MeV·cm^2^/g, Li 1.639 MeV·cm^2^/g).

### LOF prototypes irradiation and experimental results

**Figure 10:**
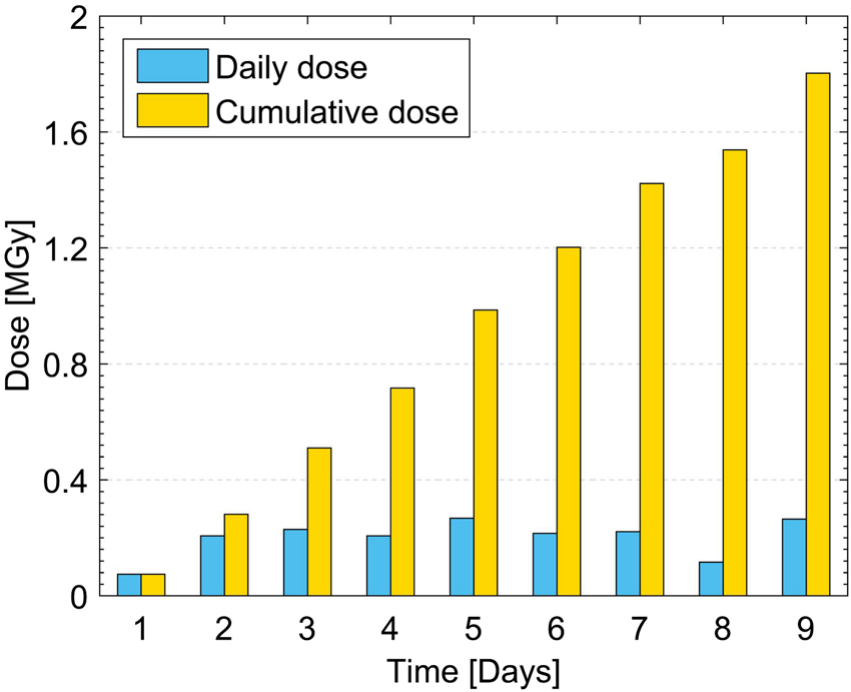
Daily and cumulative dose absorbed by the LOF devices during the exposure to proton beam.

The LOF sensors have been exposed to the 23 GeV proton beam for 9 days, with an average dose-rate of about 200 kGy/day and a total cumulated dose of about 1.8 MGy, corresponding to a total fluence of 0.67 × 10^16^ protons/cm^2^ (Fig. 10). The dose was calculated from the protons fluence evaluated through Al-foils activation measurements and normalized to 1 cm^2^. The Al-foil used for dosimetry, placed upstream the LOF sensor prototypes, is also visible in Fig. 9b,c. During the exposure, relative humidity and temperature have been monitored by using the instrumentation available at IRRAD^62^. The details on the monitoring of these parameters are shown in the Supplementary Information (Section S2).

**Figure 11:**
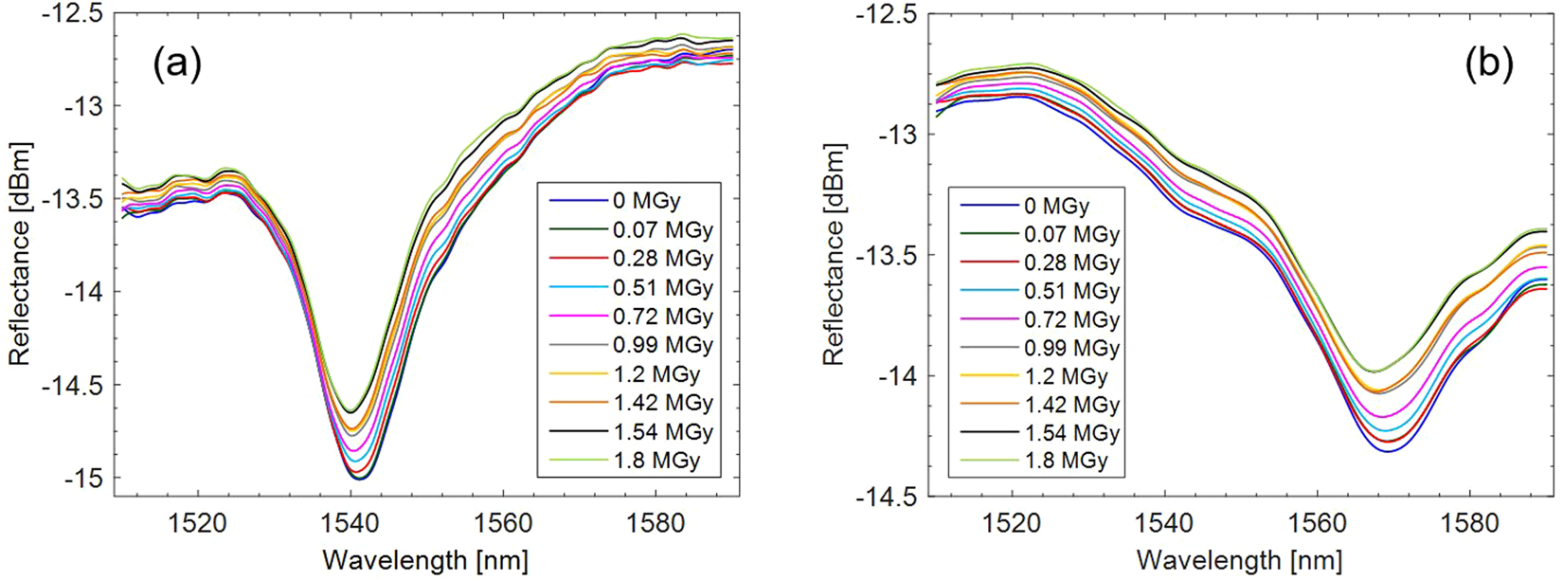
Spectra reflected by the two LOF prototypes during the exposure to 23 GeV proton beam for 9 days. The legend indicates the dose accumulated at the end of each day; **(a)** prototype A; **(b)** prototype B.

LOF prototypes were connected to a sm125 Optical Sensing Interrogator (Micron Optics Inc.) covering the wavelength range 1510–1590 nm with an accuracy of 1 pm in order to measure the effects of the proton beam on their spectral response. Spectra reflected by the LOF prototypes were saved by using a time interval of 1 hour. Because of the significant amount of data collected during the 9-days exposure and to better appreciate the spectral variations undergone by the two probes, graphics shown in Fig. 11 report only one spectrum per day, related to the dose accumulated in the previous 24 hours.

The first relevant result arising from observing the spectra shown in Fig. 11 is that the exposure to protons at high fluence levels did not produce a strong degradation of the spectrum, demonstrating the “radiation resistance” feature of the developed device. Moreover, despite the tolerances affecting the realization processes may cause slightly dissimilar spectral features, the two prototypes showed a very similar behavior, consisting of a resonance migration towards lower wavelengths.

**Figure 12:**
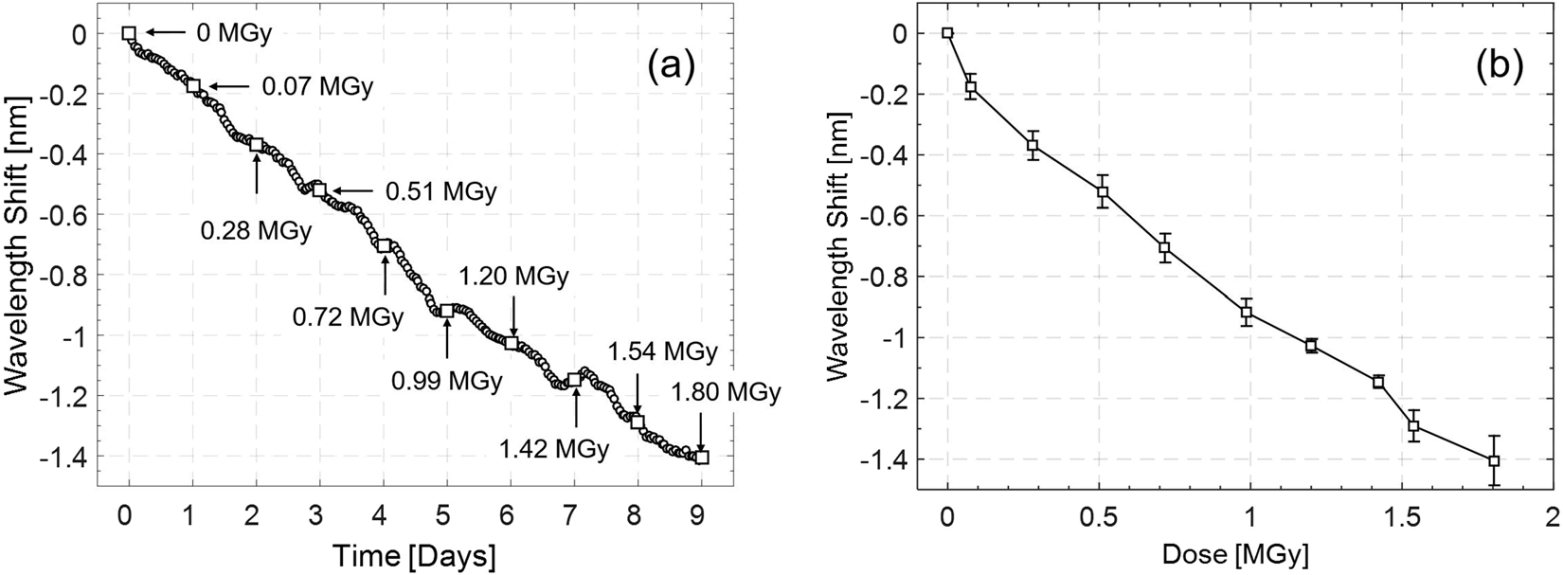
Average of the resonance wavelength shifts calculated for both LOF sensors; **(a)** wavelength shift reported as a function of the time (1 point per hour); **(b)** calibration curve displaying the wavelength shift reported as a function of the dose (1 point per day). Error bars refer to the standard deviation calculated on the two prototypes.

Based on this observation, once acquired, LOF spectra were processed by a Matlab^®^ script providing filtering and tracking of the changes occurred in the resonance wavelength. Specifically, in order to take into account also possible spectral deformations and asymmetries (mainly affecting the dips shown in Fig. 11b), an accurate approach based on the determination of the barycenter within an adequate distance (0.3 dBm) from the minimum was used. The average of the wavelength shifts undergone by the two probes with respect to the corresponding starting spectra is reported as a function of the time in Fig. 12a. Furthermore, in the same picture, the cumulative absorbed dose is indicated on a daily basis. The slightly irregular shape of the blue (negative) shift versus time could be explained by considering that the dose-rate was not constant during the exposure, as reported in Fig. 10. The calibration curve reporting the resonance wavelength shift sampled at the end of each day as a function of the cumulative absorbed dose is shown in Fig. 12b.

The average blue shift in correspondence of a total dose of 1.8 MGy was of 1.4 nm, with a sensitivity of about −0.6 nm/MGy. The slope of the calibration curve suggests that the present device could detect doses even higher than those reported here, also in view of the radiation resistance characteristic of the OF probe. In addition, the very low amplitude of the error bars, referring to the standard deviation between the responses of the two prototypes, demonstrates the high coherence exhibited by the two devices.

In order to estimate the accuracy of the proposed dosimeter, a steady state standard deviation pertaining to the resonant wavelength of 0.010 nm was calculated in time interval of 10 hours in constant environmental conditions and no radiation. By dividing this value for the sensitivity to the dose, a resolution of 16.7 kGy was achieved, corresponding to a resolution normalized to the maximum dose observed of 1.8 MGy equal to 0.9%.

Another effect resulting from the exposure to the proton beam is the progressive rise of the baseline, clearly visible from spectra shown in Fig. 11. In particular, an average variation of 0.16 dBm has been observed as response to a total dose of 1.8 MGy.

### Data analysis and discussion

Experimental results reported in the previous section have shown that the main effects of the LOF probes exposure to 23 GeV proton beam on the reflected spectrum are a blue shift of the resonance wavelength of about 1.4 nm (Fig. 12) and a slight increase of the baseline of about 0.16 dBm (Fig. 11). According to the behaviors listed in Table 3, which matches the phenomena reported in the literature with the numerical predictions, there are three parameters able to induce a blue shift as response to radiations: t_PMMA_, n_gold_ and k_fiber_. Among these, radiation changes in k_fiber_ and n_gold_ are expected to induce a decrease in the reflectance baseline in contrast with the experimental evidence of a baseline increase (see Table 3). On the contrary, a reduction of t_PMMA_ is consistent with both the trends of the resonance wavelength and of the baseline (Table 3). The only other phenomenon able to justify the experimental increase of the baseline is an enlargement of the r_holes_. Like the decrease of t_PMMA_, this too can be considered as a consequence of the compaction of the PMMA layer. However, an extension of r_holes_ is not well-matched with the experimental negative wavelength shift shown in Fig. 12. On this line of arguments, it is possible to conclude that the dominant mechanism induced by the radiation able to explain both the resonance migration to lower wavelengths and at the same time the baseline increase is the reduction of the PMMA layer thickness. Moreover, this behavior is consistent with data reported so far in the current literature^42,43^. Indeed, the PMMA presents linear polymer chains composed of monofunctional monomers. Therefore, high energy particles traversing a polymer produce bond breaking that creates reactive species, as radicals, promoting chain transfer reaction. Formation of new radicals increases with the deposited dose. As a consequence, it is possible that PMMA becomes saturated with reactive species and the PMMA exposed to ionizing dose exhibits a new morphology, leading to a compaction of the PMMA layer itself^63^.

### Conclusions and future works

The HEP community is currently preparing for the incoming upgrade of the present Large Hadron Collider, into the higher performance High Luminosity LHC, and it also envisages the possible future upgrades such as the HE-LHC and the new machines like the CLIC and the FCC. In all these options the very harsh radiation environment is evaluated to be several order of magnitudes higher than in the current LHC, demanding new dedicated technologies for both the detectors and electronics to be used. These radiation levels are expected to exceed several tens of MGy and currently available technologies for dose monitoring cannot withstand and/or measure up to these radiation levels.

An innovative dosimeter, based on the LOF technology, for ultra-high dose monitoring in the challenging scenario of the FCC at CERN has been the object of the experimental research discussed in the present paper.

In particular, we proposed here a new extrinsic OF dosimeter, based on the so-called LOF technology, consisting in a metallo-dielectric resonator realized on the OF tip including a PMMA layer.

Once identified the effects that ionizing radiation induces on the optical and geometric parameters of the LOF device, numerical simulations have been carried out to evaluate the impact induced by the variation of each single parameter on the reflected spectrum, keeping the others fixed.

Two LOF probes were designed and manufactured in order to set the resonance wavelength in the operating range of standard single mode fibers, enabling the use of robust, reliable, and commercially available optoelectronic interrogation units. Technical details were presented on selection of basic materials, manufacturing techniques and LOF probe final morphological and optical characterization. The two LOF dosimeter prototypes were exposed at the IRRAD Proton Facility at CERN in Geneva with 23 GeV protons for a total deposited dose up to 1.8 MGy in 1 cm^2^ of Si.

The exposure to protons at high dose levels did not produce any strong degradation of the spectrum, demonstrating the “radiation resistance” feature of the LOF technology. The experimental data from both prototypes show a clear variation of the LOF signal versus the total dose. The main effect consists of a resonance migration towards lower wavelengths with increasing dose level. In particular, a blue shift of the resonance wavelength of about 1.4 nm and a slight increase of the baseline of about 0.16 dBm were recorded. From the simulations, it arises that a possible reduction of the thickness of the PMMA layer of the LOF resonator (t_PMMA_) is consistent with the observed spectral trends. This hypothesis is also supported by literature^42,43^ referring to a similar behavior of PMMA exposed at different doses, energies, and radiation sources.

The preliminary experimental results reported in this paper demonstrate the effectiveness of the proposed OF device as potential dosimeter at MGy dose levels, overcoming the limit of 100 kGy achieved by the current techniques. In particular, a maximum dose of 1.8 MGy has been measured with a sensitivity of −0.6 nm/MGy and a resolution of 16.7 kGy. Additional research will follow in order to deeper understand the radiation damage mechanisms occurring in the LOF nanostructure. New LOF prototypes are being currently designed and a second irradiation campaign is being scheduled at the IRRAD Proton Facility targeting higher dose levels, to assess the LOF dosimeters performance at the tens MGy range, and then to study the post-irradiation annealing effects.

## Electronic supplementary material

Supplementary Information
