# Supplementary material for: A novel Lab-on-Fiber Radiation Dosimeter for Ultra-high Dose Monitoring
Source: Sci Rep. 2018 Dec 14;8:17841. doi: 10.1038/s41598-018-35581-3 (PMC6294823; doi:10.1038/s41598-018-35581-3)
Supplement: Supplementary file 1 — Supplementary Information [file 41598_2018_35581_MOESM1_ESM.pdf]

A novel Lab-on-Fiber Radiation Dosimeter for Ultra-high Dose Monitoring

Supplementary information

Giuseppe Quero[1,+], Patrizio Vaiano[1,+], Francesco Fienga[2,4,+], Martino Giaquinto[1], Valentina Di Meo[3], Georgi Gorine[4,5], Pierluigi Casolaro[6], Luigi Campajola[6], Giovanni Breglio[7], Alessio Crescitelli[3], Emanuela Esposito[3], Armando Ricciardi[1], Antonello Cutolo[1], Federico Ravotti[4], Salvatore Buontempo[2,4], Marco Consales[1,*], Andrea Cusano[1,*]

[1]Optoelectronics Group - Department of Engineering, University of Sannio, I-82100 Benevento, Italy
[2]Istituto Nazionale di Fisica Nucleare (INFN) - Sezione di Napoli, I-80126 Napoli, Italy
[3]Istituto per la Microelettronica e Microsistemi, Consiglio Nazionale delle Ricerche, I-80131, Napoli, Italy
[4]European Organization for Nuclear Research (CERN), 1211 Genève, Switzerland
[5]Ecole Polytechnique Federale de Lausanne (EPFL), Lausanne, Vaud, Switzerland
[6]University of Napoli Federico II, Department of Physics, I-80126 Napoli, Italy
[7]University of Napoli Federico II, Department of Electronical Engineering, I-80125 Napoli, Italy
*a.cusano@unisannio.it, *consales@unisannio.it
+these authors contributed equally to this work

S1. Additional numerical simulations

Similarly to what done for poly(methyl methacrylate) layer (in section: "LOF probe design"), the effects induced on the spectrum of the Lab-On-Fiber (LOF) resonator by the changes of the optical properties of the fiber itself and the gold layer as well as the holes radius have been investigated.
In particular, the effect expected from a perturbation of the real part of the refractive index (RI) of the fiber glass (n_{fiber}) is a linear increase of the resonant wavelength (Figure **S1.1a)**. The baseline, instead, seems to have no dependence on n_{fiber} (Figure **S1.1b**).

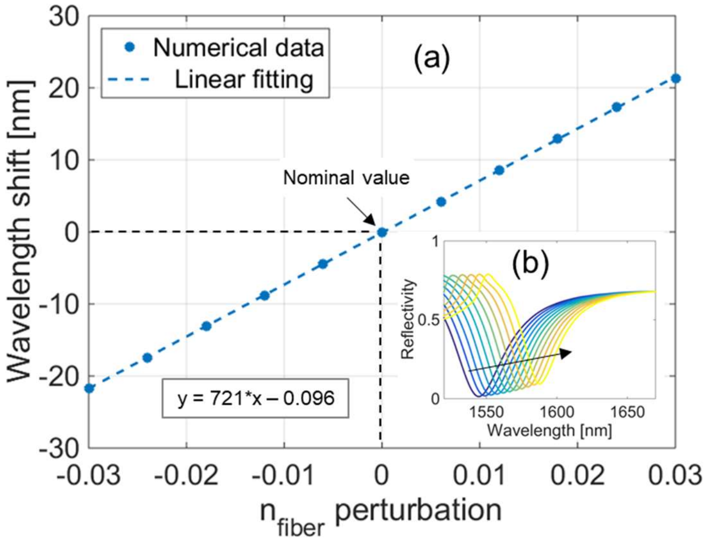

Figure S1.1: (a) Numerical simulations predicting the effects of a perturbation of n_{fiber} on the resonance wavelength. The inset box indicates the equation of the linear fitting. **(b)** Whole spectrum evolution determined by the variation of n_{fiber}. The black arrow indicates the variation direction of the parameter.

Differently, a perturbation of the imaginary part of the fiber RI (k_{fiber}) does not imply significant effects on the LOF spectrum (Figure **S1.2**), except for losses higher than 10^{-3}, when the resonance wavelength decreases (Figure **S1.2a**) and the dip starts to flatten (Figure **S1.2b**).

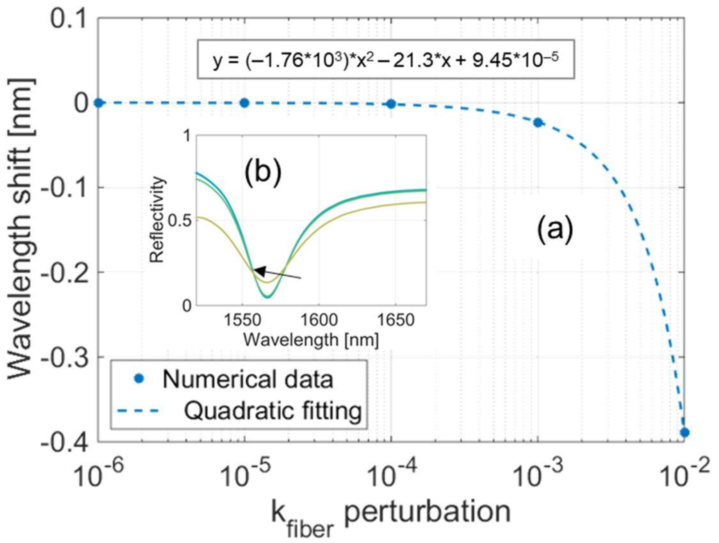

Figure S1.2: (a) Numerical simulations predicting the effects of k_{fiber} on the resonance wavelength. The inset box indicates the equation of the quadratic fitting. **(b)** Whole spectrum evolution determined by the variation of k_{fiber}. The black arrow indicates the variation direction of the parameter.

Concerning the gold layer, a perturbation of the real part of the RI (n_{gold}) is expected to induce a linear decrease of the resonance wavelength (Figure **S1.3a**). The effect of n_{gold} on the baseline is a slight decrease, as shown in Figure **S1.3b**.

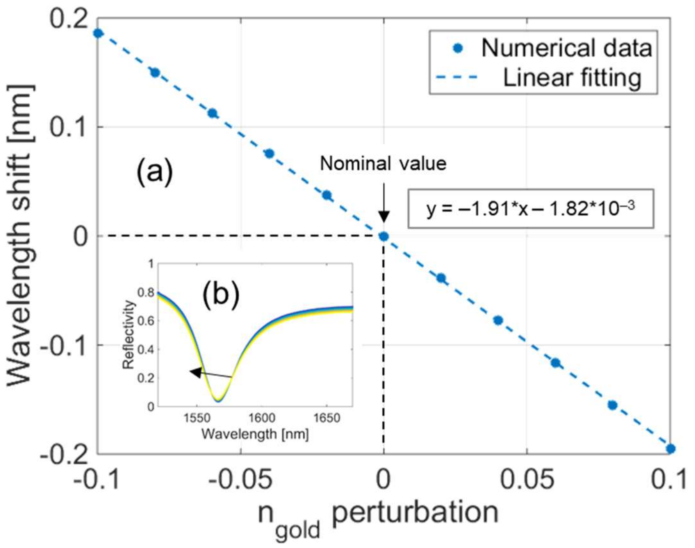

Figure S1.3: (a) Numerical simulations predicting the effects of a perturbation of n_{gold} on the resonance wavelength. The inset box indicates the equation of the linear fitting. **(b)** Whole spectrum evolution determined by the variation of n_{gold}. The black arrow indicates the variation direction of the parameter.

A perturbation of the imaginary part of the gold RI (k_{gold}) leads to a quadratic decreasing behavior of the resonance wavelength (Figure **S1.4a**). In contrast, the baseline increases with k_{gold} (Figure **S1.4b**).

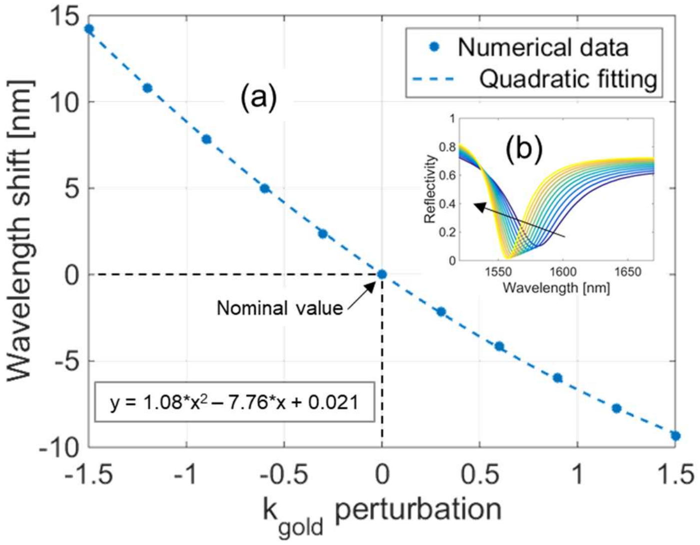

Figure S1.4: (a) Numerical simulations predicting the effects of k_{gold} on the resonance wavelength. The inset box indicates the equation of the quadratic fitting. **(b)** Whole spectrum evolution determined by the variation of k_{gold}. The black arrow indicates the variation direction of the parameter.

To conclude, an increase in the holes radius (r_{holes}) leads to a quadratic increase in the resonance wavelength (Figure **S1.5a**). Furthermore, the evolution of the whole spectrum when r_{holes} increases shows an increase of the baseline (Figure **S1.5b**).

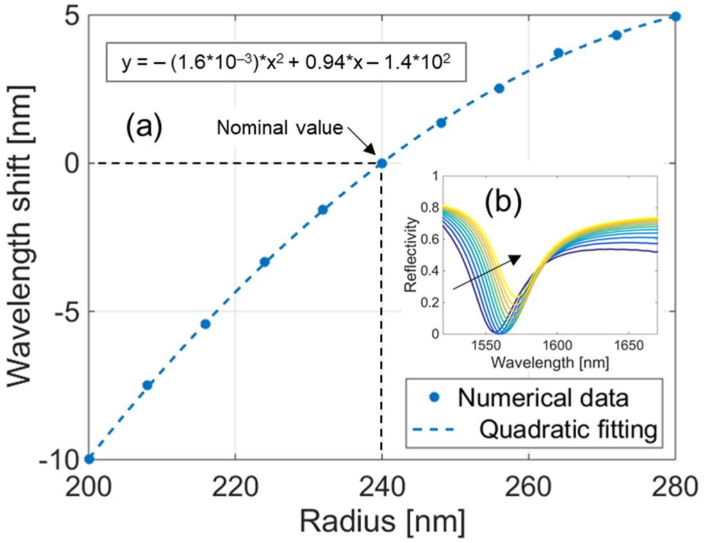

Figure S1.5: (a) Numerical simulations predicting the effects of a variation of r_{holes} on the resonance wavelength. Black dashed lines refer to nominal value of r_{holes}. The inset box indicates the equation of the quadratic fitting. **(b)** Whole spectrum evolution determined by the variation of r_{holes}. The black arrow indicates the variation direction of the parameter.

S2. Relative humidity and temperature monitoring during the experiments

During the exposure of LOF prototypes to proton beam, relative humidity and temperature have been monitored by using the instrumentation available at IRRAD facility[1,2], with a time interval of 30 minutes. In particular, data have been downloaded from the CERN website[3].

Observing Figure **S2.1**, it can be noted that the temperature remained practically unchanged during the nine days of irradiation, except for small variations contained in the range 20.9–21.5°C. Differently, relative

humidity went from values close to 60% down to values of just over 30%. The largest changes occurred in the first two days. As is evident from the comparison between Figures **S2.1** and **12**, there is no correlation between the environmental parameters and the response of the LOF devices.

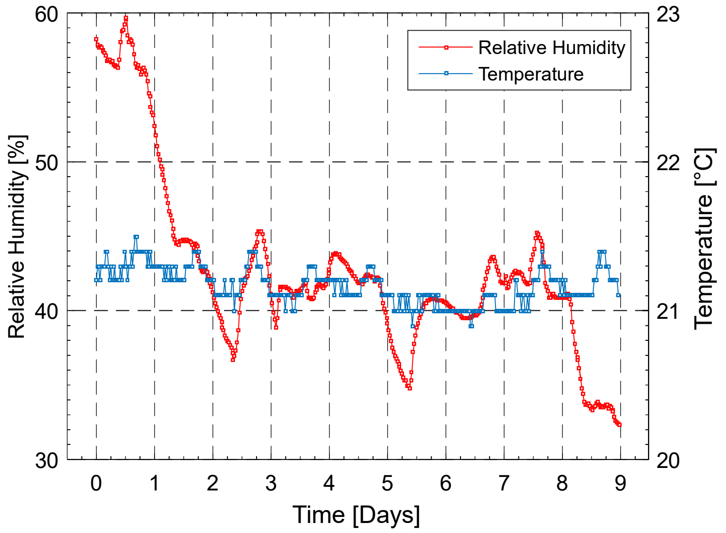

Figure S2.1: Relative humidity and temperature recorded with a time interval of 30 minutes at IRRAD during the LOF prototypes irradiation.

References

1. Gkotse, B., Moll, M., Ravotti, F. & Glaser, M. IRRAD: The New 24GeV/c Proton Irradiation Facility at CERN. *In Proc. AccApp'15*, Washington, DC, USA, 182-187 (2015).
2. Gkotse, B., Glaser, M., Matli, E., Pezzullo, G. & Ravotti, F. Environmental monitoring, control and data management system of the CERN proton irradiation facility (IRRAD). *In Proc. RADECS 2017*, Geneva, Switzerland, 2017, to be published.
3. IRRAD Environment 2016, https://ps-irrad.web.cern.ch/assets/doc/after/results/2016/DATA_IRRAD_T_HR_RUN_2016.xlsx (2018).
